# Outcomes of COMBO therapy for severe mitral regurgitation compared with transcatheter edge-to-edge repair

**DOI:** 10.3389/fcvm.2024.1223588

**Published:** 2024-02-26

**Authors:** Hiroaki Yokoyama, Tobias Friedrich Ruf, Theresa Ann Maria Gößler, Martin Geyer, Julia Zirbs, Ben Luca Schwidtal, Thomas Münzel, Ralph Stephan von Bardeleben

**Affiliations:** ^1^ Department of Cardiology and Catheterization Laboratories, Shonan Kamakura General Hospital, Kamakura, Japan; ^2^ Heart Valve Center, Department of Cardiology, Cardiology I, Universitätsmedizin Mainz, Johannes Gutenberg-University Mainz, Mainz, Germany

**Keywords:** mitral regurgitation, transcatheter mitral valve repair (TMVr), mitral transcatheter edge-to-edge therapy, M-TEER, COMBO therapy

## Abstract

**Background:**

There are different types of transcatheter mitral valve repair (TMVr) currently in clinical use, including leaflet approximation, annular cinching, and restoration of the chordal apparatus of the mitral valve (MV). While the concomitant combination (COMBO) therapy of mitral transcatheter edge-to-edge repair (M-TEER) with another TMVr concept has been proven feasible, potentially offering patient-tailored treatment for severe mitral regurgitation (MR), a comparison with M-TEER alone has not been made.

**Aims:**

To evaluate the procedural and clinical outcome of COMBO therapies compared with M-TEER alone.

**Methods:**

We included consecutive patients undergoing COMBO and M-TEER between March 2015 and April 2018 at our Heart Valve Center, while excluding patients presenting a case of redo or with previous MV surgery. Procedural outcomes and all-cause mortality were compared between COMBO therapy vs. M-TEER alone.

**Results:**

A total of 357 patients (mean age 78.9 ± 7.0 years, 53.2% male, M-TEER *n* = 322, COMBO *n* = 35; COMBO: MitraClip and the Carillon mitral contour system *n* = 26, MitraClip and Cardioband *n* = 5, and MitraClip and NeoChord *n* = 4) were analyzed. Patients with COMBO therapy had larger left chamber sizes, a lower left ventricular systolic ejection fraction (LVEF; COMBO: 37.4 ± 13.8%, M-TEER: 47.9 ± 14.3%, *p* < 0.001), and a more severe MR grade (*p* < 0.001). There were no significant differences in the prevalence of residual MR ≧2+. However, the need for re-intervention, always employing M-TEER, was more common in the COMBO group. During a mean 3.6-year long-term follow-up, there was no significant difference of all-cause mortality between both groups (Log rank *p* = 0.921).

**Conclusions:**

COMBO therapy may still be a beneficial therapy option for patients with severe MR who already have a more dilated left ventricle (LV), a more severe MR, and a more pronounced LV systolic dysfunction. The higher need for re-intervention in the COMBO group may signal more complex anatomies and possibly underlines the necessity of treating significant MR earlier. Future research is required to establish the COMBO approach as a toolbox-like treatment option, thus offering a patient-tailored approach depending on the individual anatomy and pathology.

## Introduction

Mitral regurgitation (MR) is the most common among valvular heart diseases (VHDs) (1). It has been strongly associated with decreased quality of life, increased rate of heart failure (HF) hospitalization, and shortened survival (2, 3). Mitral regurgitation can be either primary or secondary in origin. Primary MR (PMR) is related to damage to any component of the MV apparatus, i.e., chordae, leaflets, and/or papillary muscles. Secondary MR (SMR) arises from annular dilatation and tethering of the leaflets caused by a dilated and dysfunctional left ventricle (LV; vSMR) or a dilated left atrium (LA, aSMR) (4–8).

Regarding the treatment for severe MR with high surgical risk, various types of transcatheter mitral valve repair (TMVr) targeting the mitral annulus, the mitral valve (MV) chordae, as well as the MV leaflets have become feasible and safe alternatives to medical therapy and cardiac surgery (9). Especially, successful mitral transcatheter edge-to-edge repair (M-TEER) has shown to reduce mortality and HF hospitalization (10, 11). The current European Society of Cardiology (ESC) guidelines on VHD recommend M-TEER as class IIa therapy in SMR and class IIb in PMR for symptomatic severe MR patients with surgical high risk (12). There is a paucity of data on the combination of two TMVr strategies for annular and leaflet repair in one procedure only (COMBO therapy) to target the different pathophysiological components of MR (13–15). However, we recently demonstrated that COMBO therapy of TMVr is feasible and may support reverse remodeling of left cardiac chambers during 1 year after the procedure in a cohort of patients at high risk (16).

In this study, we compared the mortality and the need for re-intervention, as well as procedural and clinical outcomes between COMBO therapy and M-TEER alone for the treatment of severe symptomatic MR.

## Methods

COMBO therapy was defined as a combination of M-TEER using MitraClip NT (Abbott Laboratories, Abbott Vascular, Santa Clara, CA, USA) (17) with any other TMVr-strategy. These were a combination with either indirect annuloplasty using the Carillon mitral contour system (CMCS; Cardiac Dimensions, Kirkland, WA, USA) (18, 19) or direct annuloplasty with the Cardioband (Edwards Lifesciences, Irvine, CA, USA) (20), to improve the mitral annular dilation to control SMR, or chordal repair with the transapical NeoChord DS 1000 (NeoChord Inc., St. Louis Park, MN, USA), designed to repair PMR caused by prolapse with artificial chords (21, 22).

### Study population

Symptomatic consecutive patients presenting with severe MR and indication for transcatheter repair who underwent TMVr as single or COMBO therapeutic approach from March 2015 to April 2018 at our comprehensive Heart Valve Center were included. Redo cases, however, including both previous transcatheter and surgical interventions, as a heterogenous group were excluded for this analysis. Moreover, those cases with no comprehensive baseline echocardiography or those lost to follow-up were excluded from the cohort.

### Procedures of transcatheter mitral valve repair

**Figure 1:**
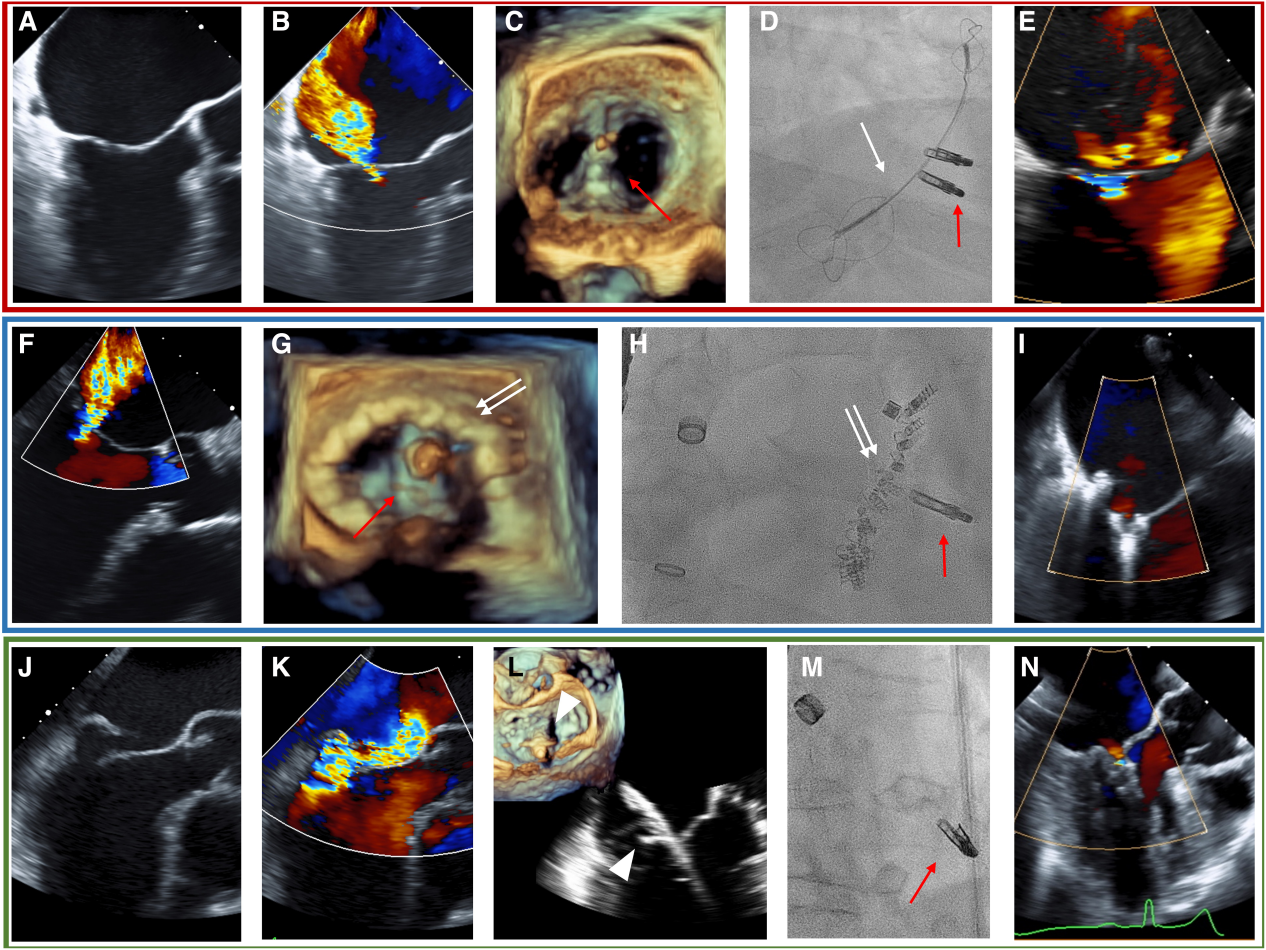
TEE and fluoroscopy (**D**, **H**, **M**) of the representative cases for each COMBO therapy using M-TEER (red arrow) with either the CMCS (white arrow) in SMR (**A–E**, red box), Cardioband (white double-arrow, blue box) in SMR (**F–I**), or NeoChord (white triangle, green box) in PMR due to posterior leaflet prolapse (**J**–**N**). (**A**) Tenting shows SMR. (**B**) Baseline MR. (**C**) 3D-echo imaging during procedure. (**D**) Fluoroscopic image during procedure. (**E**) Post-procedural MR. (**F**) Baseline MR in SMR. (**G**) 3D-echo imaging during procedure. (**H**) Fluoroscopic image during procedure. (**I**): Post-procedural MR. (**J**) Flail of posterior leaflet. (**K**) Baseline MR. (**L**) Grasping of posterior leaflet with NeoChord. (**M**) Fluoroscopic image during procedure. (**N**) Post-procedural image of MR with TEE.

The Heart Team decided to recommend the transcatheter approach over a medical or surgical pathway in each patient. The Heart Team consisted of a cardiac surgeon, an interventional cardiologist, an interventional echocardiographer, and a cardiac anesthesiologist. The COMBO therapy was discussed as an option during the Heart Team deliberation in cases where treatment of the pathology by the dedicated device was deemed difficult, e.g., large mitral valve annulus in cases that were selected for transcatheter annuloplasty, or extended prolapse in cases selected for NeoChord implantation. This option was mainly put to discussion by both the interventional team, i.e., the interventional cardiologist and the interventional echocardiographer after the patient was considered unsuitable for cardiac surgery. The decision to employ COMBO therapy was ultimately made during the procedure at the discretion of the treating interventional cardiologist. In detail, during the procedures in the COMBO therapy group, the first procedure was performed as either CMCS (Figures 1A–E, red box) or Cardioband (Figures 1F–I, blue box) in patients suffering from SMR, or NeoChord (Figures 1J–N, green box) in patients with PMR caused by Prolapse and/or flail. Each procedure was then followed by M-TEER in the same session. The technical details of each procedure have been reported previously (17–22). All procedures were performed under general anesthesia using fluoroscopy and 3D transesophageal echocardiography (TEE) guidance in all cases.

### Echocardiographic examinations

The ultrasound machines used were iE33 and Epiq7C (Philips, Andover, MA, USA), and GE Vivid E95 (GE Healthcare, Chicago, IL, USA). Images were acquired by the experienced senior cardiologists in the echocardiographic laboratory and were centrally evaluated by HY as external Corelab using IntelliSpace Cardiovascular and QLAB software (Philips). All echos analyzed were standard two-dimensional B-mode and Doppler TTE. All measurements were performed in accordance with the current recommendations of the American Society of Echocardiography (23, 24) and latest ESC VHD guidelines as well as MR and tricuspid regurgitation (TR) severity was graded according to current recommendations (12).

### Study endpoint

The outcomes were compared between COMBO therapy and M-TEER. The primary outcome was defined as all-cause mortality, while the secondary outcome was defined as the composite events of all-cause mortality and re-intervention by surgery or transcatheter methods. All-cause mortality was ascertained from the entries in patients’ health records and a central data reconciliation with the bureau of vital statistics. The census date was 31 December 2021. Furthermore, we investigated the severity of residual MR, the pressure gradient (PG) of MV at discharge, and the prevalence of re-intervention during follow-up. The study fulfills the GCP (good clinical practice) and the Declaration of Helsinki requirements and was approved by the local ethics committee (Ref. 2019-14692).

### Statistical analysis

All data endpoints were collected from records in our Heart Valve Center and the Rhineland-Palatinate bureau of vital statistics for outcome surveillance. Continuous as well as ordinally scaled variables are expressed as medians (Q_1_, Q_3_). Categorical variables are expressed as frequencies (%), using the χ^2^ test or Fisher's exact test to compare the groups. Kaplan–Meier analysis was performed using log rank test to compare the endpoint between both groups. Propensity score matching was based on age, left ventricular systolic ejection fraction (LVEF), and surgical risk as assessed by the EuroSCORE II score. A *p*-value of <0.05 was considered statistically significant. All statistical analyses were performed using IBM SPSS statistics version 27 (IBM Corp., Chicago, IL, USA) and EZR version 1.55 (Saitama Medical Center, Japan).

## Results

### Baseline patient characteristics and echocardiographic data

Between March 2015 and April 2018, 451 patients underwent M-TEER for severe MR. Of them, 38 patients with a redo case or previous surgical MV intervention, 53 patients without baseline echocardiographic parameters, and 3 patients who were lost to follow-up were excluded (Supplementary Figure S1). The remaining 357 patients (mean 78.9 ± 7.0 years, 53.2% male, M-TEER *n* = 322, COMBO therapy *n* = 35) were analyzed. Table 1 shows baseline characteristics. Patients receiving COMBO therapy were younger, had a higher weight, were less likely suffering from arterial hypertension, and had less likely already been treated with percutaneous coronary intervention (PCI). There were no differences in medication and laboratory data. The calculated surgical risk was elevated for the whole cohort [EuroSCORE II 4.9 (Q1–Q3: 3.4, 7.3)], and similar in-between groups [M-TEER 5.0 (3.5, 7.6) vs. COMBO 4.5 (3.2, 6.1), *p* = 0.305].

**Table 1:** Baseline characteristics.

	Overall *n* = 357	M-TEER *n* = 322	COMBO *n* = 35	*p*-value
Demographics				
Age, years	80.0 [75.0, 83.0]	80.0 [76.0, 84.0]	76.0 [73.0, 79.5]	<0.001
Sex (male), *n* (%)	190 (53.2)	167 (51.9)	23 (65.7)	0.153
Height, mm	169.0 [164.0, 175.0]	168.0 [164.0, 175.0]	170.0 [167.5, 176.0]	0.185
Weight, kg	74.0 [65.0, 82.0]	73.5 [65.0, 81.0]	76.0 [68.0, 88.5]	0.120
BMI, kg/m^2^	25.7 [23.6, 28.0]	25.6 [23.7, 27.8]	27.0 [23.5, 29.4]	0.27
BSA, m^2^	1.83 [1.72, 1.98]	1.83 [1.72, 1.97]	1.92 [1.75, 2.02]	0.089
NYHA functional class, *n* (%)				
I	2 (0.6)	2 (0.6)	0 (0.0)	0.477
II	57 (16.0)	52 (16.2)	5 (14.3)	
III	215 (60.4)	190 (59.2)	25 (71.4)	
IV	82 (23.0)	77 (24.0)	5 (14.3)	
	(*n* = 356)	(*n* = 321)		
Past medical history				
Hypertension, *n* (%)	316 (88.8)	291 (90.4)	25 (73.5)	0.007
Diabetes mellitus, *n* (%)	97 (27.2)	89 (27.6)	8 (22.9)	0.69
Chronic kidney disease, *n* (%)	175 (49.0)	158 (49.1)	17 (48.6)	0.999
Atrial fibrillation, *n* (%)	267 (74.8)	237 (73.6)	30 (85.7)	0.151
COPD, *n* (%)	49 (13.7)	47 (14.6)	2 (5.7)	0.198
Pacemaker/ICD/CRT, *n* (%)	97 (27.2)	84 (26.1)	13 (37.1)	0.166
Previous PCI, *n* (%)	223 (62.6)	209 (65.1)	14 (40.0)	0.005
Previous CABG, *n* (%)	58 (16.2)	53 (16.5)	5 (14.3)	0.999
Previous stroke, *n* (%)	28 (7.8)	27 (8.4)	1 (2.9)	0.338
Medication				
Beta blocker, *n* (%)	291 (81.7)	261 (81.3)	30 (85.7)	0.648
RAS inhibitor, *n* (%)	285 (80.1)	255 (79.4)	30 (85.7)	0.505
Diuretics agents, *n* (%)	335 (94.1)	303 (94.4)	32 (91.4)	0.448
MRA, *n* (%)	282 (79.0)	253 (78.3)	29 (82.9)	0.555
Laboratory data				
BNP, pg/ml	545.0 [269.5, 1,081.0]	563.0 [273.0, 1,095.0]	359.0 [239.5, 819.3]	0.089
Creatinine, mg/dl	1.31 [1.01, 1.79]	1.32 [1.01, 1.81]	1.21 [0.97, 1.48]	0.222
Troponin I, pg/ml	20.5 [10.7, 42.6]	19.9 [10.9, 43.4]	24.2 [8.3, 35.7]	0.85
Risk assessment				
EuroSCORE II	4.9 [3.4, 7.3]	5.0 [3.5, 7.6]	4.5 [3.2, 6.1]	0.305

BNP, brain natriuretic peptide; BMI, body mass index; BSA, body surface area; CABG, coronary artery bypass graft; COPD, chronic obstructive pulmonary disease; CRT, cardiac resynchronization therapy; ICD, implantable cardioverter defibrillator; MRA, mineralocorticoid receptor antagonist; PCI, percutaneous coronary intervention; RAS, renin-angiotensin system.

Values are median [Q1, Q3] or *n* (%).

Baseline echocardiographic parameters are summarized in Table 2. Patients receiving COMBO therapy had a greater dilatation of the LA, as well as of the LV [LA volume (LAV)_COMBO_ 118.6 ± 52.8 ml vs. LAV_M−TEER_ 83.2 ± 60.0 ml (*p* = 0.001), LV end-systolic volume (LVESV)_COMBO_117.6 ± 80.9 ml vs. LVESV_M−TEER_ 66.1 ± 43.9 ml (*p* < 0.001), LV end-diastolic volume (LVEDV)_COMBO_: 174.6 ± 95.8 ml vs. LVEDV_M−TEER_ 118.8 ± 53.3 ml; *p* < 0.001]. Furthermore, LV dysfunction was also more severe in the COMBO group when compared with patients receiving M-TEER (LVEF_COMBO_ 37.4 ± 13.8% vs. LVEF_M−TEER_ 47.9 ± 14.3%; *p* < 0.001). Moreover, patients in the COMBO therapy group had more severe MR grades [MR grade_COMBO_ 3 (3, 4) vs. MR grade_M−TEER_ 3 (3, 3); distribution of MR grade 4+: COMBO 45.7% vs. M-TEER 18.0%; *p* = 0.001].

**Table 2:** Baseline echocardiographic parameters.

	Overall *n* = 357	M-TEER *n* = 322	COMBO *n* = 35	*p*-value
LVESD, mm	38.0 [30.0, 50.0]	37.5 [29.0, 49.0]	50.0 [42.3, 59.3]	<0.001
LVEDD, mm	55.0 [47.0, 62.0]	53.0 [46.0, 61.0]	61.5 [56.3, 67.0]	<0.001
LVESV, ml	58.7 [36.5, 94.9]	54.4 [35.2, 88.5]	107.5 [61.0, 162.7]	<0.001
LVESV index, ml/m^2^	30.8 [19.9, 49.1]	28.9 [19.5, 46.7]	48.8 [29.5, 79.1]	<0.001
LVEDV, ml	112.7 [81.2, 154.3]	109.6 [80.0, 146.6]	164.7 [102.8, 218.1]	<0.001
LVEDV index, ml/m^2^	61.9 [45.9, 81.5]	60.7 [45.5, 79.0]	82.5 [58.8, 106.5]	<0.001
LVEF, %	47.5 [34.9, 58.1]	48.7 [36.4, 59.1]	34.9 [26.3, 47.9]	<0.001
LVEF index, %/m^2^	25.6 [18.3, 32.7]	26.3 [19.4, 33.0]	18.3 [13.7, 24.3]	<0.001
IVSD, mm	10.1 [8.7, 11.6]	10.0 [8.8, 11.6]	10.4 [8.2, 12.0]	0.897
PWD, mm	9.7 [8.7, 11.0]	9.8 [8.9, 11.1]	8.9 [8.2, 9.7]	<0.001
LV mass, g	209.7 [158.5, 266.3]	206.9 [156.9, 261.3]	250.2 [205.3, 294.7]	0.007
LV mass index, g/m^2^	111.8 [90.9, 142.6]	110.5 [90.0, 140.4]	125.4 [102.3, 155.3]	0.027
LAV, ml	74.9 [59.0, 97.7]	72.1 [57.5, 95.2]	102.1 [81.7, 150.7]	<0.001
LAV index, ml/m^2^	41.6 [32.3, 52.6]	40.3 [31.5, 51.6]	50.0 [42.1, 77.1]	<0.001
MV annulus diameter, mm	36.0 [32.0, 39.0]	35.0 [32.0, 38.0]	40.0 [36.5, 42.0]	<0.001
Etiology of MR				
PMR/mix, *n* (%)	132 (37.0)	124 (38.5)	8 (22.9)	0.096
SMR, *n* (%)	225 (63.0)	198 (61.5)	27 (77.1)	
MR grade, *n* (%)				
1+	0 (0.0)	0 (0.0)	0 (0.0)	0.001
2+	41 (11.5)	39 (12.1)	2 (5.7)	
3+	242 (67.8)	225 (69.9)	17 (48.6)	
4+	74 (20.7)	58 (18.0)	16 (45.7)	
MR vena contracta, mm	6.8 [6.0, 7.6]	6.8 [5.9, 7.6]	7.3 [6.4, 7.8]	0.046
TR grade, *n* (%)				
0–1+	150 (42.3)	131 (40.9)	19 (54.3)	0.677
2+	110 (31.0)	101 (31.6)	9 (25.7)	
3+	78 (22.0)	72 (22.5)	6 (17.1)	
4+	11 (3.1)	10 (3.1)	1 (2.9)	
5+	6 (1.7)	6 (1.9)	0 (0.0)	
	(*n* = 355)	(*n* = 320)		

LVESD, left ventricular end-systolic diameter; LVEDD, left ventricular end-diastolic diameter; LVESV, left ventricular end-systolic volume; LVEDV, left ventricular end-diastolic volume; IVSD, interventricular septum diameter; PWD, posterior wall diameter; LV mass, left ventricular mass.

Values are median [Q1, Q3], or *n* (%).

### Primary and secondary outcomes

**Figure 2:**
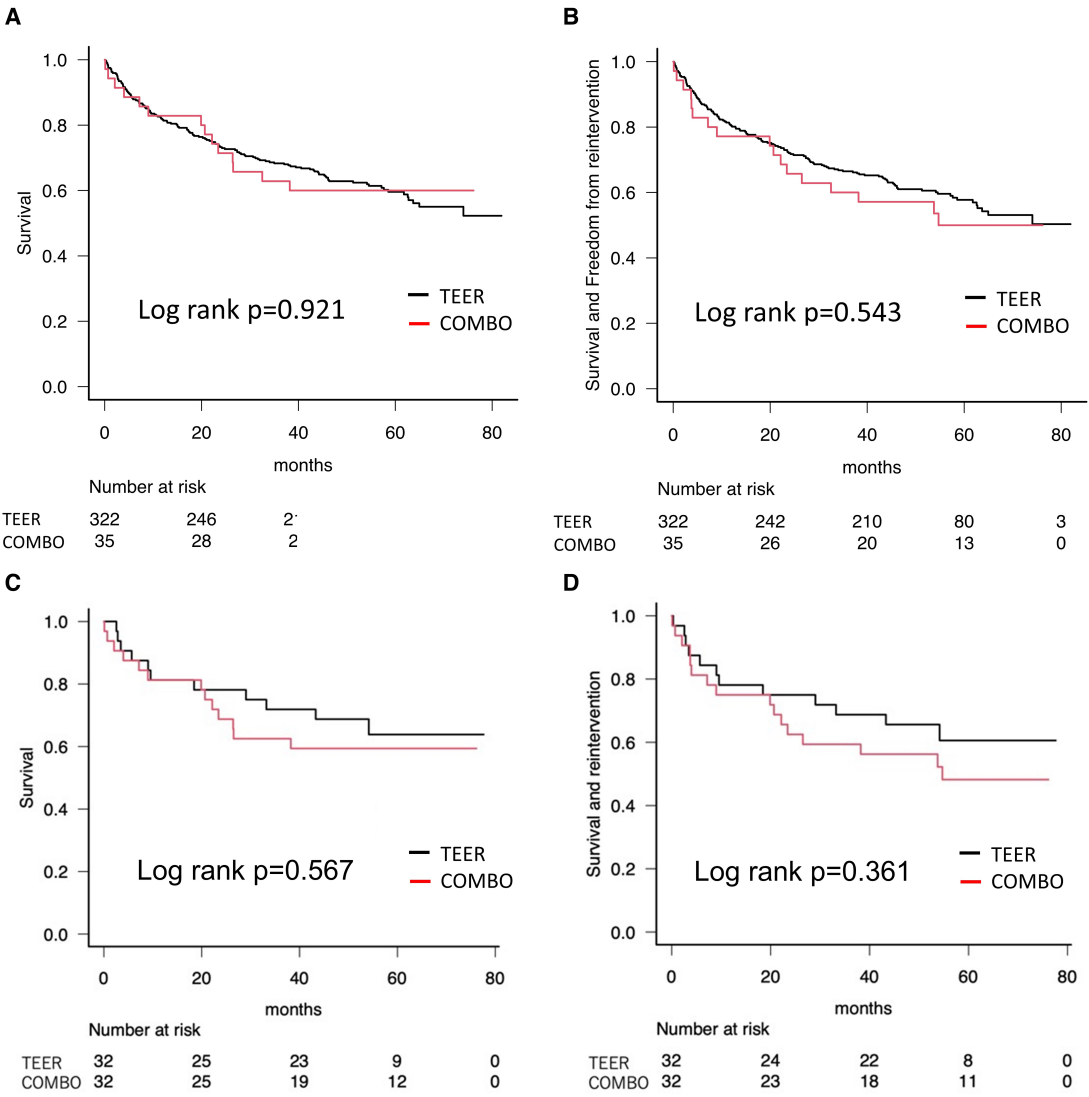
Kaplan–Meier curves showing survival. (**A**) All-cause mortality as a comparison between COMBO therapy and M-TEER. (**B**) All-cause mortality and re-intervention as a comparison between COMBO therapy and M-TEER. (**C**) Propensity score matched all-cause mortality. (**D**) Propensity score matched combined endpoint of all-cause mortality and re-intervention.

Based on the census date of 31 December 2021, the mean long-term follow-up duration in our cohort was 1,310 ± 711 days (COMBO; 1,307 ± 707 days, M-TEER; 1,332 ± 761 days, *p* = 0.847). Figure 2 shows the Kaplan–Meier curves about the survival and the composite events of survival and re-intervention as a comparison between COMBO therapy and M-TEER. Survival rate in all patients was 94.3% [95% confidence interval (CI): 79.0–98.5] at 30 days, 82.9% (95% CI: 65.8–91.9) after 1 year, 71.4% (95% CI: 53.4–83.5) after 2 years, and 62.9% (95% CI: 44.8–76.5) after 3 years (Figure 2A). There was no significant difference of survival and the composite endpoint of survival and re-intervention between both the groups (Log rank *p* = 0.921 and 0.543, respectively) (Figures 2A,B). Finally when using propensity score matching, neither M-TEER nor COMBO showed a difference in all-cause mortality (Log rank *p* = 0.567, Figure 2C) or the combined endpoint (Log rank *p* = 0.361, Figure 2D). The re-intervention was always M-TEER.

Supplementary Figure S2 reveals the Kaplan–Meier curves about the survival and the composite events of survival and re-intervention according to the etiology of MR, comparing both groups, with no significant difference in-between groups (Log rank *p* = 0.74, Log rank *p* = 0.643, respectively). Patients with COMBO therapy also had no significant difference in all-cause mortality and the composite events of survival and re-intervention according to the etiology of MR (Log rank *p* = 0.652 and 0.082, respectively; Supplementary Figure S3). As the rate of re-intervention seemed to be higher in patients for PMR using COMBO therapy (Supplementary Figure 2B), we conducted a separate analysis of each etiology. Here, there was also no difference in mortality when comparing the outcome of PMR (*p* = 0.409) vs. SMR (*p* = 0.443) (Supplementary Figure S4).

### Procedure characteristics and outcomes

The procedure characteristics and follow-up in all COMBO therapy patients are demonstrated in Supplementary Table S1. Patients were treated with MitraClip and CMCS, MitraClip and Cardioband, and MitraClip and NeoChord (*n* = 26, *n* = 5, and *n* = 4, respectively). Twenty-five patients in M-TEER received another concomitant transcatheter valve intervention (aortic valve or tricuspid valve) and 15 patients received iatrogenic atrial septal defect closure at the end of the procedure for residual significant right-to-left shunt with deoxygenation, while no patient in COMBO therapy receiving any of the two additional treatments. There was no difference in the number of MitraClip NT used between both the groups (COMBO; 1.6 ± 0.7, M-TEER; 1.6 ± 0.6, *p* = 0.945). Short-term safety also showed no significant differences between groups (Supplementary Table S2). In detail, there were 7 (2%) overall in-hospital deaths [M-TEER *n* = 6 (1.9%) vs. COMBO *n* = 1 (2.9%), *p* = 0.517], 1 (0.3%) cardiac tamponade, and 3 (0.8%) strokes, each in the M-TEER group (*p* = 0.999, each). The median time from procedure to discharge was 5 (4, 7) days in all patients [M-TEER 5 (4, 6) vs. COMBO 5 (4, 9), *p* = 0.359].

There was no difference in the post-interventional MV PG at discharge [COMBO 3.1 ± 1.5 mmHg (34/35), M-TEER 3.5 ± 1.6 mmHg (308/322), *p* = 0.250]. While not statistically significant, there was a trend toward higher prevalence of residual MR ≧2+ at discharge in the COMBO group [COMBO 34.3% (12/35) vs. M-TEER 20.6% (65/315), *p* = 0.083]. During follow-up, four patients were referred for redo M-TEER in the COMBO group, significantly more than in the M-TEER group (COMBO 11.4%, M-TEER 2.5%, *p* = 0.022).

**Figure 3:**
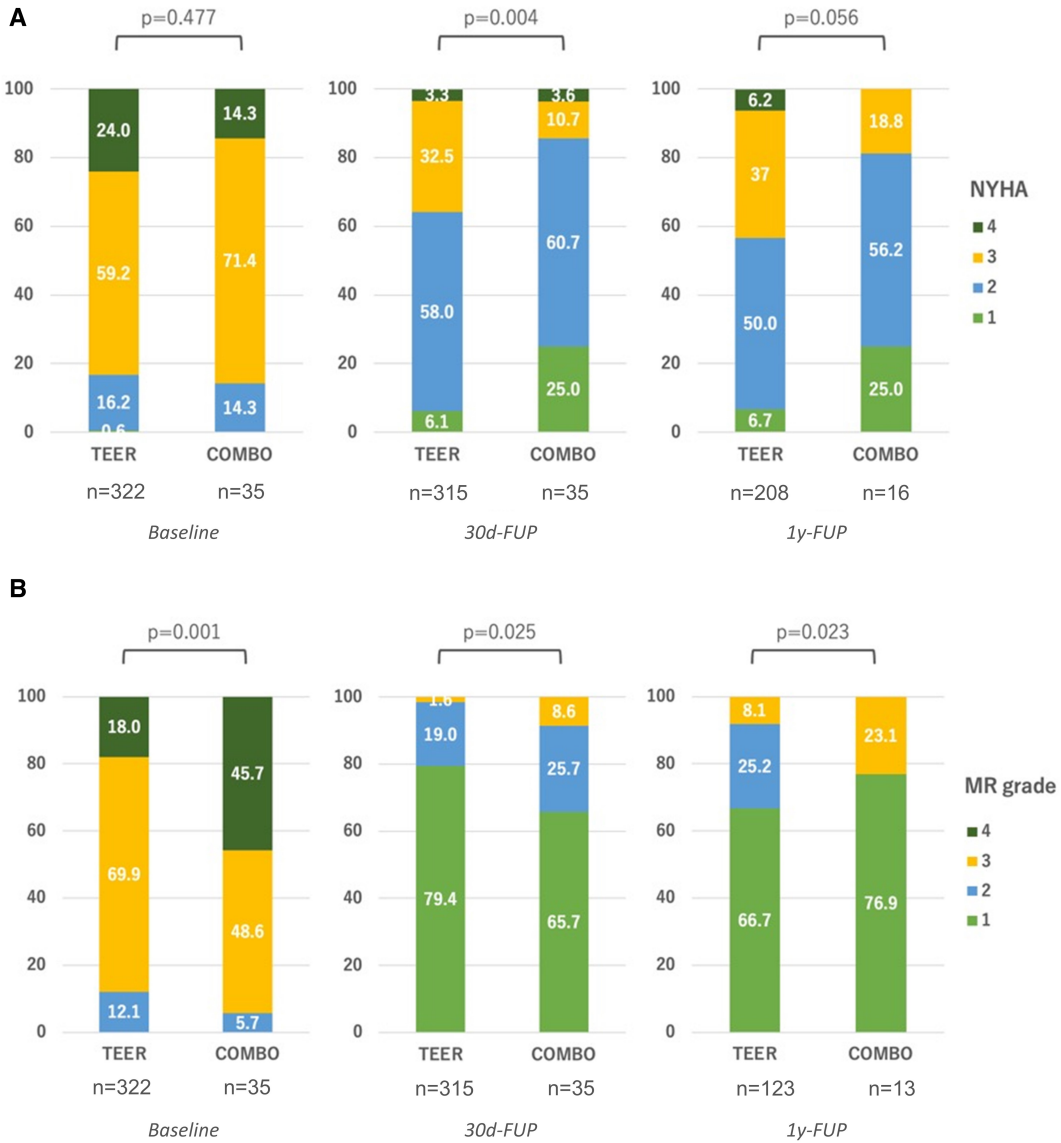
Clinical results during follow-ups. (**A**) Development of NYHA functional class between groups; (**B**) Development of residual MR between groups. FUP, follow-up.

Clinically, we observed a shift toward better New York Heart Association (NYHA) functional classes in each group. While the NYHA functional class was statistically not significant in-between groups at baseline, at 30-day follow-up (*p* = 0.004), as well as at 1-year follow-up or later (*p* = 0.056), the groups differed with an apparent greater gain for the COMBO group (Figure 3A). Similarly, all groups saw a decrease in MR over time, while the differences in baseline MR grade favoring the M-TEER group were sustained (Figure 3B).

## Discussion

This study demonstrates that COMBO therapy appears as feasible in a patient collective more severely affected by MR grade, chamber dilatation, and systolic dysfunction as in patients treated with M-TEER alone: we found no significant difference in short-term safety parameters, in all-cause mortality, and in the composite events of all-cause mortality and re-intervention compared with M-TEER, independent of the etiology of MR. This is further highlighted by the long follow-up with a mean of 3.6 years.

There was a higher need for re-intervention in the COMBO group, and a tendency to higher residual MR grades. This comes as no surprise, as the baseline MR grades were higher in the COMBO therapy group, as well as the dimensions and volumes of the left-sided heart chambers. This suggests a disease stage in the COMBO treatment group less amendable to MV repair, as enlarged left-sided heart dimensions, as well as increased severity of MR grade, have been shown to be predictors of unsuccessful MV repair (25). This is further underlined by the finding that this difference in MR grade was sustained over time.

Also, general differences in baseline characteristics suggest a sicker patient population. Despite these differences, the survival rate was comparable between the groups. Aside from the obvious limitations of this study discussed in what follows, one reason might be that the population of the COMBO therapy group was younger, also having a lower likelihood of arterial hypertension and previous PCI. The LVEF was significantly lower by ≥10%, arising from more dilated LVs in systole and diastole. In HF populations, dilation of the LV has earlier been identified as an independent contributor to a poor prognosis in patients with or without myocardial infarction (26, 27). MV interventions for SMR may not lead to better outcomes in HF patients with enlarged LV dimensions (28, 29). In surgical annuloplasty for SMR, the outcome is poor when the baseline LV end-diastolic diameter exceeds 65 mm (28). Furthermore, not just LV dimensions but also the relationship between LV dimensions and MR severity should be considered. The relatively novel framework of proportionality in SMR patients was devised from the insights gained from the MitraFR and COAPT trials (11, 29, 30). In MitraFR, M-TEER did not lead to preferable clinical and functional outcomes with larger LV volumes and lesser MR grade [proportionate MR: LVEDV index 135 ± 35 ml/m^2^, effective regurgitant orifice area (EROA) 31 ± 10 mm^2^] (31). Conversely, in COAPT, with smaller LV volumes and more MR (disproportionate MR: LVEDV index 101 ± 34 ml/m^2^, EROA 41 ± 15 mm^2^), M-TEER had a significant effect on the clinical and survival outcomes starting at 1 year, and remaining stable up to 5 years (30). This indicates that treatment of SMR may not be effective when the left ventricle is too large (32). These anatomic and functional parameters can characterize the determinants of successful SMR treatment to help the decision-making in clinical practice (31, 33). In this context, COMBO therapy itself in our study may show the need for more intensive therapy in a population that “came in late” on a temporal and disease progression scale. Yet, if these patients would have had even less favorable results when treated with M-TEER only remains a speculation.

Three-quarters of the patients in the COMBO therapy group were treated with the CMCS combined with M-TEER. With respect to the CMCS, Anker et al. reported that even patients with severely enlarged LV diameters experienced LV reverse remodeling and reduced hospitalization rates for HF 1 year after the procedure (32). Hence, one might draw the clinical implication from our findings that COMBO therapy—especially with M-TEER and CMCS—might be effective in patients even with enlarged LV volumes not amenable for an M-TEER alone strategy. We cannot know whether these patients could have achieved similar results with one therapy alone, as Cardioband, CMCS, and NeoChord show improvements of MR, and there are vast data demonstrating effectiveness of M-TEER. However, since the baseline characteristics demonstrate that the COMBO group had larger LA and LV volumes, larger mitral annulus diameters, lower LVEF, and a more severe MR, all predictors of worse outcome, COMBO therapy shows effectiveness even in these difficult cases.

The M-TEER devices were those available during 2015–2018. Since then, newer iterations have been developed, with the MitraClip XTW of the fourth generation being larger and wider than the MitraClip NT that was used most often in this study. One might argue that fewer patients would therefore require COMBO therapy, with larger M-TEER devices possibly being more effective. Conversely, COMBO therapy could also be used in more patients that were considered not treatable before, and furthermore, COMBO therapy itself could be more effective using the larger M-TEER devices, especially since Cardioband and CMCS already offer a wide range of device sizes to fit different, i.e., larger anatomies, while a COMBO therapy with NeoChord and larger M-TEER devices in PMR could be used to address cases with especially pronounced leaflet redundancy.

The survival rate after M-TEER was 73.2% at 5 years in the operable EVEREST trial with PMR and SMR, 57.2% at 3 years in the COAPT trial with SMR only, and 66.1% at 2 years in the MitraFR trial with advanced progression SMR patients (10, 11, 29). Looking at the 5-year COAPT data, the initial result favoring treatment with M-TEER remained stable with all-cause mortality at 42.7% in the device group and 32.8% in the control group (30). The survival rate after CMCS implantation was 67.9% at 3 years (*n* = 74) (34). With respect to Cardioband, the survival rate was reported as being 87% at 1 year (*n* = 60) in SMR, and with respect to NeoChord, 94.0% at 3 years (*n* = 203, in pure PMR) (35, 36).

COMBO therapy can likely increase procedure risks in a surgical high-risk population. Our findings indicate that patients with COMBO therapy that had no direct procedure-related complications seem to have a favorable short-term and long-term mortality compared with the patients treated with M-TEER.

The gains over time in NYHA functional class were consistent throughout both groups, as can be expected with any therapy addressing symptomatic MR. The differences found between groups at the two follow-up intervals, apparently favoring the COMBO therapies, must be carefully interpreted. On the one hand, the patients in the COMBO group seemed sicker, as mentioned. Therefore, although there was no difference in NYHA functional class at baseline, this group might have had “more to gain” clinically, especially as MR seemed more severe in this group while almost all patients of both the groups were in NYHA functional class III or even IV, anyway. On the other hand, this interpretation is subject to the obvious limitations of this study.

Our study demonstrates that in patients with more enlarged LV, more profound systolic impairment, and more severe MR grades, COMBO therapy can provide meaningful results. Therefore, by paying attention to the functional and anatomic parameters of the individual patient characteristics, our treatment toolbox is enhanced.

## Limitations

There are several limitations to this study: Its design is retrospective and observational from a single center, so hidden confounders could be present. Furthermore, the number of patients treated with COMBO therapy is small. Therefore, especially small differences between the study groups might have been missed. The procedures were carried out in a dedicated, large volume Heart Valve Center, making results possibly not generalizable. We excluded three foreign patients that were lost to follow-up, who were not listed in the bureau of vital statistics. This could possibly cause a selection bias, when the clinical outcome was death. Yet, because the patients were all from the M-TEER alone group, the impact would most likely be small.

Then, while the COMBO approach was suggested as an option during the Heart Team deliberations, the ultimate decision was made by the interventionalist during the procedure. This most likely caused a significant selection bias as was discussed previously: the patients in the COMBO group suffered from more pronounced disease, but other confounders might also be present.

The follow-up rate of 38% for M-TEER, and 45% for COMBO, at 1 year was only moderate, calling the validity of the data somewhat into question. Then, it might be argued that the effectiveness of the different strategies might be better analyzed using propensity matching based on anatomical parameters, such as mitral pathology, indices of left atrial and left ventricular size and function. However, with the limitations already mentioned, especially the retrospective design, only moderate follow-up rate and without a core lab to offer a uniform echocardiographic analysis, a propensity score matching would not be feasible. However, while this study is certainly not powered to detect fine significant differences in mortality, and performance of propensity score matching in small sample sizes shows reduced performance (37), the analysis based on age, LVEF, and surgical risk still adds information to demonstrate the feasibility of the COMBO approach in selected patients. Furthermore, since the data on mortality was gathered from census, this somewhat compensates for the only moderate follow-up adherence.

Changes of medical drug therapy treatment for heart failure after the procedure were not documented like in more rigorous randomized clinical trials. This could have an impact on the results of this study. Also, SGLT-2 inhibitors were not administered for HF in this group, because approval for this medication to treat patients suffering from heart failure with reduced ejection fraction (HFrEF) was granted in late 2020.

Because the field of interventions in structural heart disease is evolving quickly, extrapolation of our findings to the latest generation of devices, e.g., M-TEER (Edwards Pascal P5, or Abbott MitraClip fourth generation), or future generations, e.g., annuloplasty devices, may not be possible.

This is not a randomized study. The improvement in outcomes seen for patients with more severe MR and larger chamber sizes in the COMBO group shows that COMBO therapy seems effective and safe, but not superior to M-TEER. In fact, the insights of this study are intended as “proof of concept,” demonstrating that physicians may need to use a toolbox to individualize patient treatment, rather than to be confined to a “one size fits all” solution.

This study did not focus on PMR or SMR alone. Moreover, the COMBO therapy group consisted of three different devices combined with M-TEER for the sample volume leading to some heterogeneity. However, the patients with COMBO therapy with two devices other than M-TEER, for example the CMCS and NeoChord, were excluded from this study, and the human experience with the COMBO therapy so far is limited in registries or publications.

To fully appreciate the potential of COMBO therapy, and to make a deeper impact on clinical practice, prospective—and preferably randomized—clinical research is required in the future, each focusing on one COMBO approach. Since the patients included in this study showed severe comorbidities and challenging anatomies and/or pathologies, a reasonable choice could be to include symptomatic patients suffering from severe MR that show already significant dilatation of the mitral annulus. When investigating NeoChord, the patients suffering from PMR caused by extensive prolapse and flail of one leaflet would be considered, whereas in SMR, these patients, also showing significant dilatation of the LV and pronounced tethering of the leaflets, would most likely be logical choices for treatment with either CMCS or Cardioband.

We did not analyze the cost-effectiveness of the different treatment strategies. Therefore, COMBO therapy might not be as feasible as desired, especially when local reimbursement is limited or restricted.

In consequence, this study's findings should be interpreted with caution and as “hypothesis generating.”

## Conclusions

This study showed that, compared with M-TEER alone, there were no significant differences in safety outcomes. Yet, patients treated with COMBO therapy suffered from more impaired LVEF, more severe MR, and larger ventricles at baseline. Although the re-intervention rate was higher in this higher-risk patient population, promising results could be obtained. This demonstrates that COMBO therapy is feasible and safe, possibly offering a toolbox to individualize patient treatment. The higher need for re-intervention in the COMBO group possibly underlines the need to treat severe MR earlier in smaller ventricles. However, future research is required to establish the COMBO approach as such a toolbox-like treatment option.

## Impact on daily practice

Combination (COMBO) therapy, which refers to the therapeutic strategy with more than two TMVr devices, is rarely used as a treatment for patients with severe MR. However, we compared this procedure with M-TEER, which is now a most common TMVr for severe MR, and found that COMBO therapy might be beneficial even in patients who already had a more dilated LV and a more pronounce LV systolic dysfunction. Now various TMVr devices are available for the treatment of severe MR in clinical use, offering a toolbox with its uses determined by the background and the anatomy of the patient.

## Data Availability

The datasets presented in this article are not readily available because of local data security restrictions (Datenschutzbeauftragter, University Medicine Mainz). Requests to access the datasets should be directed to tobias.ruf@unimedizin-mainz.de.

## Supplementary material

Supplementary Figure S1, Supplementary Figure S2, Supplementary Figure S3, Supplementary Figure S4, Supplementary Table S1, Supplementary Table S2 Patient flow chart. MR, mitral regurgitation; M-TEER, mitral transcatheter edge-to-edge repair. Kaplan–Meier curves according to etiology of MR. (**A**) All-cause mortality as a comparison between COMBO therapy and M-TEER. (**A**) All-cause mortality and re-intervention as a comparison between COMBO therapy and M-TEER. MR, mitral regurgitation; PMR, primary MR; SMR, secondary MR; M-TEER, mitral transcatheter edge-to-edge repair. The Kaplan–Meier curves in COMBO therapy according to etiology of MR. (**A**) All-cause mortality. (**A**) All-cause mortality and re-intervention. MR, mitral regurgitation; PMR, primary MR; SMR, secondary MR; M-TEER, mitral transcatheter edge-to-edge repair. The Kaplan–Meier curves in each MR etiology. (**A**) SMR. (**B**) PMR. M-TEER, mitral transcatheter edge-to-edge repair. Procedural characteristics and outcomes. In-hospital safety.
