# Supplementary material for: Outcomes of COMBO therapy for severe mitral regurgitation compared with transcatheter edge-to-edge repair
Source: Front Cardiovasc Med. 2024 Feb 26;11:1223588. doi: 10.3389/fcvm.2024.1223588 (PMC10925764; doi:10.3389/fcvm.2024.1223588)
Supplement: Supplementary Figure S1, Supplementary Figure S2, Supplementary Figure S3, Supplementary Figure S4, Supplementary Table S1, Supplementary Table S2 — Patient flow chart. MR, mitral regurgitation; M-TEER, mitral transcatheter edge-to-edge repair. Kaplan–Meier curves according to etiology of MR. (A) All-cause mortality as a comparison between COMBO therapy and M-TEER. (A) All-cause mortality and re-intervention as a comparison between COMBO therapy and M-TEER. MR, mitral regurgitation; PMR, primary MR; SMR, secondary MR; M-TEER, mitral transcatheter edge-to-edge repair. The Kaplan–Meier curves in COMBO therapy according to etiology of MR. (A) All-cause mortality. (A) All-cause mortality and re-intervention. MR, mitral regurgitation; PMR, primary MR; SMR, secondary MR; M-TEER, mitral transcatheter edge-to-edge repair. The Kaplan–Meier curves in each MR etiology. (A) SMR. (B) PMR. M-TEER, mitral transcatheter edge-to-edge repair. Procedural characteristics and outcomes. In-hospital safety. [file Presentation1.pdf]

Supplemental Material

Supplemental Figures

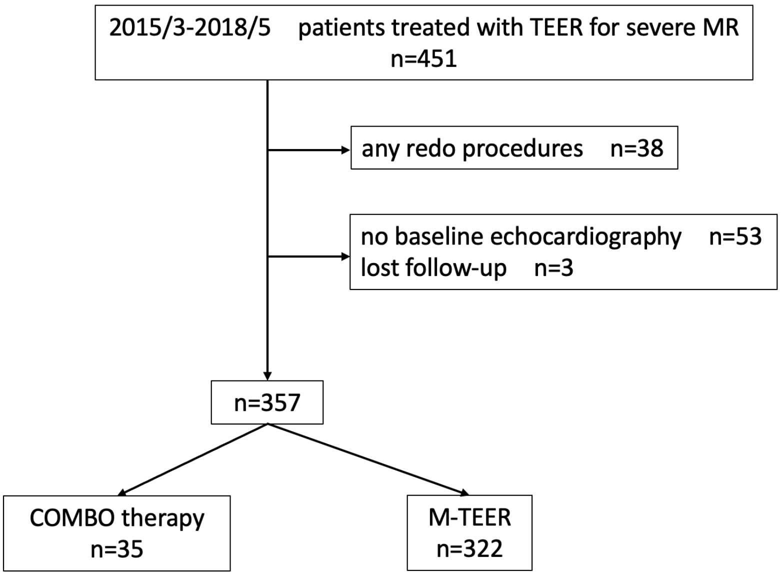

Supplemental Figure 1. Patient flow chart.

Abbreviations: MR; mitral regurgitation, M-TEER; transcatheter edge-to-edge repair for MR.

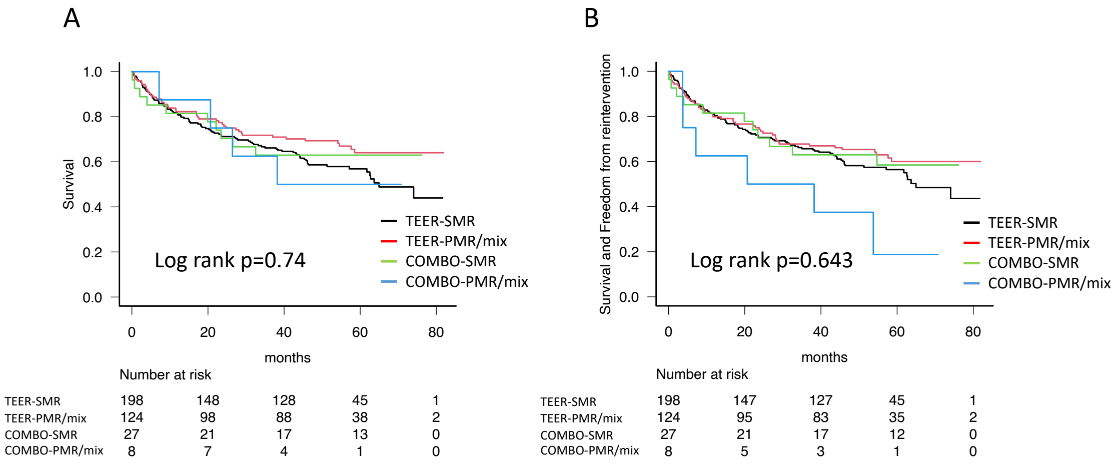

Supplemental Figure 2. Kaplan Meier curves according to etiology of MR.

A. All-cause mortality in comparison between COMBO therapy and M-TEER. B. All-cause mortality and re-intervention in comparison between COMBO therapy and M-TEER. Abbreviations: MR; mitral regurgitation, PMR; primary MR, SMR; secondary MR, M-TEER; transcatheter edge-to-edge repair for MR.

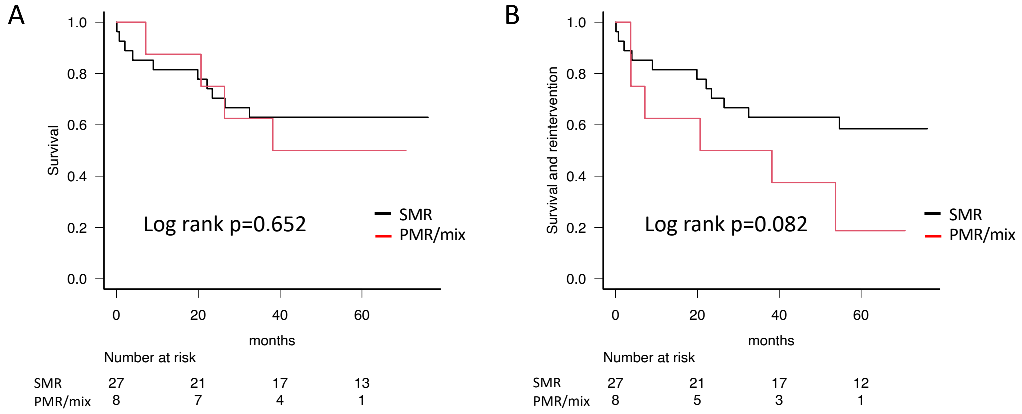

Supplemental Figure 3. The Kaplan Meier curves in just COMBO therapy according to etiology of MR.

A. All-cause mortality. B. All-cause mortality and re-intervention.

Abbreviations: MR; mitral regurgitation, PMR; primary MR, SMR; secondary MR, M-TEER; transcatheter edge-to-edge repair for MR.

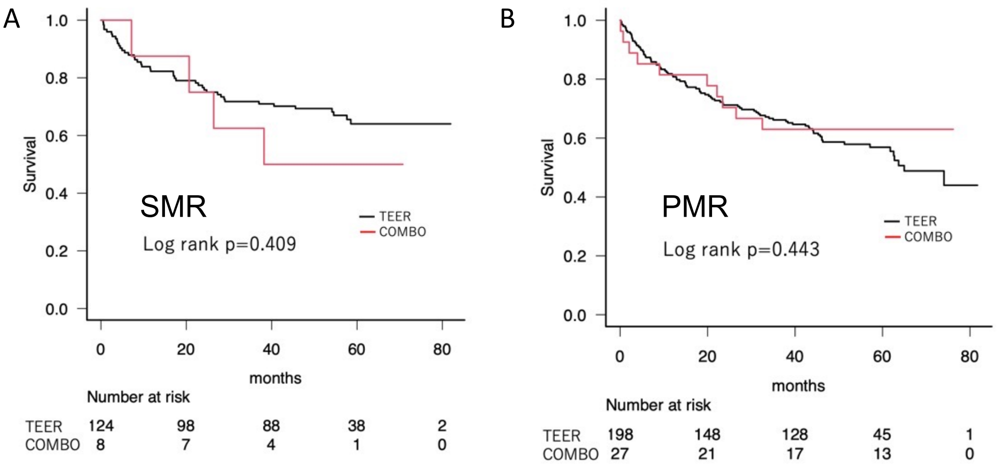

Supplemental Figure 4. The Kaplan Meier curves in each MR eteiology.

Abbreviations: M-TEER; transcatheter edge-to-edge repair for MR.

Supplemental Tables

Supplemental Table 1

Procedural characteristics and outcomes

Patient	Age, years	Logistic EuroScore, %	LVEF, %	MV annulus diameter, mm	Pre MR grade	Etiology of MR	Device	Post MR grade	Post MV PG, mmHg
**1**	79	Unknown	30.5	44	4	SMR	M-TEER 2 Clips Carillon 14×20×50 mm	2	Unknown
**2**	80	12.49	46.8	34	3	PMR	M-TEER 2 Clips Carillon 11×20×80 mm	3	2
**3**	73	31	24.3	45	2	SMR	M-TEER 1 Clip Carillon 9×18×60 mm	1	2
**4**	77	22.62	40.8	42	4	SMR	M-TEER 1 Clip Carillon 11×80×20 mm	3	2
**5**	80	23.64	36.0	42	3	SMR	M-TEER 1 Clip Carillon 12×20×80 mm	1	5
**6**	63	16.34	27.7	36	3	SMR	M-TEER 1 Clip Carillon 9×18×60 mm	2	2
**7**	81	16.56	56.7	36	3	SMR	M-TEER 2 Clips Carillon 13×18×70 mm	1	5
**8**	79	50.1	58.3	35	4	PMR	M-TEER 1 Clip	1	6

							Carillon 7×18×60 mm		
**9**	80	Unknown	32.4	33	4	SMR	M-TEER 2 Devices Carillon 12×18×60 mm	2	1
**10**	79	14.8	54.8	36	3	SMR	M-TEER 1 Device Carillon 10×20×60 mm	1	2
**11**	76	18.78	25.0	40	4	SMR	M-TEER 2 Devices Carillon 14×20×60 mm	2	7
**12**	79	18.35	32.8	37	3	SMR	M-TEER 2 Devices Carillon 10×18×60 mm	1	4
**13**	82	56.2	34.3	33	4	SMR	M-TEER 1 Device Carillon 12×20×60 mm	2	3
**14**	73	21.09	24.5	41	3	SMR	M-TEER 2 Devices Carillon 13×20×70 mm	1	2
**15**	76	20	34.9	42	3	SMR	M-TEER 2 Devices Carillon 8×18×80 mm	1	4
**16**	42	8.96	21.6	37	3	SMR	M-TEER 3 Devices Carillon 11×20×80 mm	1	6
**17**	67	Unknown	36.8	35	3	SMR	M-TEER 2 Devices	1	3.3

							Carillon 13×20×70 mm		
**18**	58	8.96	22.1	39	3	SMR	M-TEER 3 Devices Carillon 12×20×60 mm	1	3
**19**	54	11.07	27.7	37	3	SMR	M-TEER 1 Device Carillon 12×18×60 mm	1	3
**20**	74	11.98	49.4	49	3	SMR	M-TEER 2 Devices Carillon 10×20×80 mm	1	2
**21**	74	22.22	32.0	42	2	SMR	M-TEER 2 Devices Carillon 12×20×80 mm	1	2
**22**	77	17.41	19.0	39	4	SMR	M-TEER 1 Device Carillon 11×16×60 mm	2	1.6
**23**	69	19.96	16.4	39	4	SMR	M-TEER 2 Devices Carillon 11×18×80 mm	1	2
**24**	84	19.86	35.6	45	3	SMR	M-TEER 3 Devices Carillon 13×20×80 mm	1	4
**25**	74	Unknown	34.7	39	4	SMR	M-TEER 2 Devices Carillon 12×20×60 mm	1	3
**26**	73	13.87	48.9	40	4	PMR	M-TEER 2 Devices	3	6

							Carillon 12×20×80 mm		
**27**	79	19.34	44.8	43	4	PMR	M-TEER 2 Devices<br>4 Neochord	2	4
**28**	80	11.78	60.9	41	4	PMR	M-TEER 2 Devices<br>3 Neochord	2	3
**29**	84	10.11	63.4	31	4	PMR	M-TEER 1Devices<br>2 Neochord	1	2.6
**30**	79	11.85	65.1	45	3	PMR	M-TEER 1 Devices<br>4 Neochord	1	2
**31**	72	15.43	49.0	40	3	SMR	M-TEER 1 Device<br>Cardioband 14 ancors	1	2
**32**	69	18.45	19.2	38	3	SMR	M-TEER 1 Device<br>Cardioband 17 ancors	1	3
**33**	85	Unknown	41.9	46	4	PMR	M-TEER 1 Device<br>Cardioband 17 ancors	1	3
**34**	73	10.4	19.3	40	4	SMR	M-TEER 1 Device<br>Cardioband 14 ancors	1	2
**35**	73	13.13	40.1	45	4	SMR	M-TEER 1 Device<br>Cardioband 17 ancors	2	2

Abbreviation: LVEF; left ventricular ejection fraction, M-TEER; transcatheter edge-to-edge repair for MR, MR; mitral regurgitation, MV; mitral valve, PG; pressure gradient, PMR; primary mitral regurgitation, SMR; secondary mitral regurgitation.

Supplemental Table 2

In hospital safety

	Overall	M-TEER	COMBO	p
in-hospital death, n (%)	7 (2.0)	6 (1.9)	1 (2.9)	0.517
cardiac tamponade, n (%)	1 (0.3)	1 (0.3)	0 (0.0)	0.999
stroke, n (%)	3 (0.8)	3 (0.9)	0 (0.0)	0.999
emergent surgical conversion, n (%)	0 (0.0)	0 (0.0)	0 (0.0)	NA
days from procedure to discharge, days	5 [4, 7]	5 [4, 6]	5 [4, 9]	0.359

Values are median [Q1, Q3], or n (%).

Abbreviation: M-TEER; transcatheter edge-to-edge repair for MR, MR; mitral regurgitation
